# GSTM3, but not IZUMO1, is a cryotolerance marker of boar sperm

**DOI:** 10.1186/s40104-019-0370-5

**Published:** 2019-08-05

**Authors:** Marc Llavanera, Ariadna Delgado-Bermúdez, Beatriz Fernandez-Fuertes, Sandra Recuero, Yentel Mateo, Sergi Bonet, Isabel Barranco, Marc Yeste

**Affiliations:** Biotechnology of Animal and Human Reproduction (TechnoSperm), Unit of Cell Biology, Department of Biology, Institute of Food and Agricultural Technology, University of Girona, C/Maria Aurèlia Campany, 69, Campus Montilivi, E-17003 Girona, Spain

**Keywords:** Boar, Cryopreservation, GSTM3, IZUMO1, ROS, Sperm

## Abstract

**Background:**

Cryopreservation is currently the most efficient method for long-term preservation of mammalian gametes and is extensively used in swine artificial insemination (AI) centres. However, it is well-known that cryopreservation procedures induce changes in the water phase in both intra and extracellular compartments, which alter the content and localisation of several proteins and ends up curtailing the structural integrity of functional sperm (i.e., cryoinjuries). Alterations and deficiencies of sperm-oocyte binding proteins during gamete recognition are one of the causes of reproductive failure both *in vitro* and *in vivo*. In this sense, characterisation of cryopreservation effects upon oocyte-binding proteins of sperm, such as IZUMO1 and GSTM3, is essential when assessing the impact of this technique in swine reproduction.

**Results:**

Cryopreservation was found to induce changes in the localisation of IZUMO1 and GSTM3 in boar sperm. However, the relative content of both proteins was not altered after thawing. Furthermore, whereas IZUMO1 content was found not to be related to the cryotolerance of boar sperm, GSTM3 content was observed to be higher in poor (PFE) than in good (GFE) freezability ejaculates in both pre-frozen (1.00 INT·mm^2^ ± 0.14 INT·mm^2^ vs. 0.72 INT·mm^2^ ± 0.15 INT·mm^2^; *P* < 0.05) and post-thawed (0.96 INT·mm^2^ ± 0.20 INT·mm^2^ vs. 70 INT·mm^2^ ± 0.19 INT·mm^2^; *P* < 0.05) samples. Moreover, GSTM3 levels were found to be higher in those spermatozoa that exhibited low mitochondrial activity, high reactive oxygen species (ROS) production, and high membrane lipid disorder post-thaw (*P* < 0.05).

**Conclusions:**

The difference in GSTM3 content between GFE and PFE, together with this protein having been found to be related to poor sperm quality post-thaw, suggests that it could be used as a cryotolerance marker of boar spermatozoa. Furthermore, both IZUMO1 and GSTM3 relocate during cryopreservation, which could contribute to the reduced fertilising capacity of frozen-thawed boar sperm.

**Electronic supplementary material:**

The online version of this article (10.1186/s40104-019-0370-5) contains supplementary material, which is available to authorized users.

## Introduction

Sperm cryopreservation is currently the most efficient method for long-term storage of mammalian gametes for artificial insemination (AI). In spite of this, freezing and thawing processes are known to harm spermatozoa (i.e., cryoinjuries) because of the phase change of water in both intracellular and extracellular compartments [1]. Cryoinjuries cause detrimental effects on sperm motility and plasma membrane integrity. They also lead to changes in sperm protein levels, localisation, function and tyrosine-phosphorylation; alterations of mitochondrial function, and high ROS production, among many others [2]. This wide range of cryoinjuries impair sperm function and survival, underlying a significant decrease in the reproductive performance after thawing [1]. Furthermore, boar sperm are more susceptible to damage by the freeze-thaw process than sperm from other species [3], which leads to a decrease in the use of this technique for swine sperm preservation.

It is well known that boar sperm plasmalemma is highly sensitive to temperature changes, due to the abundance of unsaturated phospholipids and to the low amount of cholesterol [4]. As a result, temperatures lower than or equal to 5 °C lead to the destabilisation of sperm plasma membrane [2]. In turn, this leads to protein translocation and/or loss of function, thereby being a potential cause of subfertility in frozen-thawed sperm [2]. In pigs, as in other species, differences in the ability to withstand freeze-thawing processes have been found between ejaculates. This has led ejaculates to be classified as good freezability ejaculates (GFE) or poor freezability ejaculates (PFE) [5, 6].

Among other factors, male subfertility has been associated to genetic abnormalities [7] and low levels of sperm-oocyte binding proteins [8]. For successful fertilisation to occur, sperm must be able to bind and penetrate the zona pellucida (ZP) of the oocyte, and then bind and fuse to the oocyte plasma membrane. It is evident then that any alteration that inhibits sperm from carrying out these processes would result in male subfertility or infertility. Several sperm proteins, such as ADAM family members [9], PH-20 [10], TMEM95 [11], IZUMO1 [12] and GSTM3 [13, 14], have been shown to play an essential role for oocyte binding. Thus, improper expression and/or localisation of such proteins due to cryopreservation procedures is likely to lead to subfertility because of a failure in oocyte recognition. Two essential fertility-related proteins of boar sperm, which may be altered by cryopreservation procedures and in consequence impair sperm fertilising ability, are IZUMO1 and GSTM3.

IZUMO1 is a member of the immunoglobulin superfamily (IgSF) which was discovered for the first time in mouse as an essential sperm-oocyte binding protein [12] through the interaction with its oocyte receptor JUNO (Folate receptor 4, FOLR4) after acrosome reaction [15]. While studies in bull sperm by Fukuda et al. [16] showed no changes in the relative IZUMO1-content in response to cryopreservation, they reported an aberrant translocation of this protein to the whole equatorial or acrosomal regions in acrosome-exocytosed sperm, resembling to the pattern observed in *in vitro* capacitated and acrosome-reacted sperm. In pigs, Kim et al. [17] reported that IZUMO1 is mainly located at the equatorial segment and inner acrosomal membrane of capacitated sperm.

On the other hand, glutathione S-transferase Mu 3 (GSTM3) is a member of a large group of cytosolic, membrane-bound multi-gene and multi-functional isoenzymes that catalyse a number of reduced glutathione-dependent reactions which are involved in cellular protection against oxidative stress and toxic chemicals [18]. It is known that Mu members of the glutathione S-transferase family are attached to the sperm plasma membrane via non-covalent interactions and their activity is mainly restricted to the plasma membrane rather than other compartments (e.g. mitochondria) [13, 19]. In mammalian sperm, membrane-bound GSTM3 is involved in the prevention of oxidative stress [20] and in the fertilisation of the oocyte through its interaction with ZP4 [14]. Kwon and colleagues [21] found that, in boar sperm, higher levels of GSTM3 are associated to smaller litter sizes. Moreover, Kumar et al. [22] showed that cryopreservation reduces GSTM3-content and induces its relocation from the connecting, mid, principal and end pieces, to the mid-piece in buffalo sperm after freeze-thawing.

Despite the clear role IZUMO1 and GSTM3 on sperm fertility, there is no literature available on their localisation pattern in fresh boar sperm, nor on the effects of cryopreservation upon their relative content and localisation. Thus, this work sought to elucidate the effects of cryopreservation on the presence, content and localisation of IZUMO1 and GSTM3 in boar sperm, as this may contribute to our understanding of the reduced fertility of frozen-thawed sperm. In addition, the ability of these proteins to serve as predictors of sperm cryotolerance was also explored.

## Materials and methods

### Boars and ejaculates

Twelve different ejaculates from different sexually mature Piétrain boars (*n* = 12) were purchased from an AI centre (Grup Gepork S.L., Masies de Roda, Spain). Ejaculates were collected using the gloved-hand method, diluted 1:2 (*v:v*) using a commercial extender (Vitasem LD; Magapor S.L., Zaragoza, Spain), packed in bags and transported at 17 °C to the laboratory within 5 h post-collection.

Upon arrival, each ejaculate was split into four aliquots. The first one was used to assess pre-frozen sperm quality, whereas the second and third aliquots were used for Western blot and immunofluorescence analysis, respectively. Finally, the fourth aliquot was stored at 17 °C until its cryopreservation the following day.

### Sperm cryopreservation

The fraction intended for cryopreservation was processed following the protocol described by Prieto-Martínez et al. [23], with minor modifications. Briefly, samples were split into 50 mL tubes and centrifuged at 2400×*g* at 15 °C for 3 min. Then, supernatants were discarded and sperm pellets were pooled and resuspended to a final concentration of 1.5 × 10^9^ spermatozoa per mL with lactose-egg yolk freezing medium (LEY; 80% (*v:v*) lactose [0.31 mol/L; Sigma-Aldrich, St. Louis, MO, USA], and 20% (*v:v*) egg yolk). Samples were cooled down to 5 °C for 120 min and diluted to a final concentration of 1 × 10^9^ spermatozoa per mL with LEYGO medium (6% glycerol [Sigma-Aldrich] and 1.5% Orvus ES paste [Equex STM; Nova Chemical Sales Inc., Scituate, MA, USA]). Then, sperm were loaded into 0.5 mL straws (Minitub Ibérica, S.L.; Tarragona, Spain) and placed in a controlled-rate programmable freezer (Icecube 14SB; Minitub Ibérica, S.L.). Cooling rates and times were those used by Casas et al. [6]: 100 s from 5 °C to − 5 °C at a rate of − 6 °C per min, 113 s from − 5 °C to − 80 °C at a rate of − 39.82 °C per min, 30 s at − 80 °C (no temperature variation), and 70 s from − 80 °C to − 150 °C at a rate of − 60 °C per min. Finally, straws were plunged into liquid nitrogen (− 196 °C) and stored.

For thawing, three straws per ejaculate were placed into a water bath at 38 °C with vigorous shaking for 10 s. The straw content was diluted 1:3 with pre-warmed Beltsville Thawing Solution (BTS) [24]. Finally, frozen-thawed samples were placed at 38 °C and sperm quality was assessed after 30 and 240 min of incubation. Additionally, Western blot and immunofluorescence analysis of cryopreserved sperm were performed 30 min post-thaw.

### Flow cytometry analyses

Four sperm parameters (plasma membrane integrity, sperm membrane lipid disorder, mitochondrial membrane potential and intracellular levels of superoxides [O_2_^−^▪]) were evaluated. All sperm samples were diluted with phosphate buffered saline 1× (PBS) to a final concentration of 5 × 10^6^ cells per mL in a final volume of 0.6 mL before they were stained with the corresponding protocol. The flow cytometry assessments were conducted using a Cell Laboratory QuantaSC cytometer (Beckman Coulter; Fullerton, CA, USA), and samples were excited with an argon ion laser (488 nm) set at a power of 22 mW. A total of three technical replicates, with a minimum of 10,000 events per replicate, were evaluated for each ejaculate and sperm parameter. Flowing Software (Ver. 2.5.1; University of Turku, Finland) was used to perform flow cytometric data analysis, following the recommendations of the International Society for Advancement of Cytometry (ISAC). The corresponding mean ± standard error of the mean (SEM) was subsequently calculated. Sperm viability was evaluated by assessing their membrane integrity using the SYBR14/PI according to the protocol of Garner and Johnson [25]. Membrane lipid disorder of pre-frozen and frozen-thawed sperm was evaluated by M540 and YO-PRO-1 co-staining, following the procedure of Rathi et al. [26] with minor modifications by Yeste et al. [27]. Mitochondrial membrane potential of pre-frozen and frozen-thawed sperm was evaluated following a protocol modified from Ortega-Ferrusola et al. [28], assessed through JC-1 staining. Finally, sperm oxidative stress was evaluated by assessing intracellular levels of hydrogen superoxides (O_2_^−^▪) through co-staining with HE and YO-PRO-1, following a modification of the procedure described by Guthrie and Welch [29]. All protocols are described in detail in Additional file 1.

### Sperm motility

Sperm motility was evaluated using a commercial computer assisted sperm analysis (CASA) system consisting of a phase contrast microscope (Olympus BX41) at 100× magnification (Olympus 10× 0.30 PLAN objective lens; negative phase-contrast field) connected to a computer equipped with ISAS software (Integrated Sperm Analysis System V1.0; Proiser, Valencia, Spain). Three replicates per sample, with a minimum of 1000 spermatozoa per replicate, were assessed placing 5 μL of each sperm sample onto a pre-warmed Makler counting chamber (Sefi-Medical Instruments, Haifa, Israel). The recorded sperm motility parameters provided by the software were sperm progressive motility (PMOT, %); curvilinear velocity (VCL, μm/s); average path velocity (VAP, μm/s); straight line velocity (VSL, μm/s); amplitude of lateral head displacement (ALH, μm); beat cross frequency (BCF, Hz); linearity (LIN, %); and straightness (STR, %). A sperm cell was considered to be motile when VAP was higher than 10 μm/s. The corresponding mean ± SEM was subsequently calculated.

### Western blot analysis

Pre-frozen and frozen-thawed boar sperm were used for Western blot analysis. Samples were centrifuged and resuspended in lysis buffer. Following this, samples were incubated in agitation at 4 °C for 30 min. After incubation, all samples were sonicated thrice, centrifuged at 10,000×g and stored at − 80 °C prior to protein quantification. Quantification of total protein in all samples was carried out in triplicate by a detergent compatible (DC) method (BioRad).

Ten micrograms of total protein were resuspended in Laemmli reducer buffer 2× and boiled at 96 °C before proteins were loaded onto the upper stacking gel. After, electrophoretic protein separation, proteins from the gel were transferred onto polyvinyl fluoride membranes (Immobilion-P; Millipore, Darmstadt, Germany) using Trans-Blot® Turbo™ (BioRad). Blocked membranes were then incubated overnight with primary antibodies: anti-IZUMO1 polyclonal rabbit antibody (ref. NBP1–83086; Novus Biologicals, Littleton, CO, USA; 1:10,000; *v:v*) or anti-GSTM3 polyclonal rabbit antibody (ref. ARP53561_P050; Aviva Systems Biology, San Diego, USA; 1:20,000; *v:v*). Next, membranes were washed and incubated with secondary goat anti-rabbit antibody conjugated with horseradish peroxidase (HRP; Dako, Derkman A/S; Denmark) for an hour with agitation (1:15,000 (*v:v*) dilution for IZUMO1 and 1:25,000 (*v:v*) for GSTM3). Finally, membranes were washed and bands were visualised with a chemiluminescent substrate (ImmobilionTM Western Detection Reagents, Millipore) and scanned with G:BOX Chemi XL 1.4 (SynGene, Frederick, MT, USA).

Following these steps, the membranes were stripped by incubation with agitation at room temperature with a stripping buffer. Next, stripped membranes were blocked and then incubated overnight with anti-alpha-tubulin monoclonal mouse antibody (ref. MABT205, Millipore; 1:100,000, *v:v*). Thereafter, membranes were washed and incubated with secondary anti-mouse HRP–conjugated polyclonal rabbit antibody (ref. P0260; Dako; 1:150,000, *v:v*) for 1 h. Finally, membranes were washed, incubated with Immobilon Western Chemiluminescent HRP Substrate (Millipore) and scanned with G:BOX Chemi XL 1.4 (SynGene).

Three technical replicates per sample were evaluated and bands were quantified using Quantity One Version 4.6.2 software package (BioRad). Pattern quantifications were normalized using alpha-tubulin, and the corresponding mean ± SEM of each sample was subsequently calculated.

The specificity of primary antibodies was confirmed through peptide competition assays utilising IZUMO1- (ref. NBP1-83086PEP; Novus Biologicals) and GSTM3- (ref. AAP53561; Aviva Systems Biology) immunising peptides, 20 times in excess with regard to their respective antibodies (see Additional file 2). Detailed Western blot protocol is described in Additional file 1.

### Immunofluorescence

Localisation of IZUMO1 and GSTM3 in pre-frozen and frozen-thawed boar sperm was evaluated through immunofluorescence. Sperm samples were diluted to a final concentration of 3 × 10^6^ cells per mL, fixed with 1.5% (*w:v*) paraformaldehyde and washed with PBS 1×. Two drops per sample were placed onto different slides, and all slides were blocked and permeabilised with blocking solution. Then, all samples were incubated overnight with primary antibodies anti-IZUMO1 polyclonal rabbit antibody (ref. NBP1–83086; Novus Biologicals, Littleton, CO, USA; 1:250; *v:v*) and anti-GSTM3 polyclonal rabbit antibody (ref. ARP53561_P050; Aviva Systems Biology; 1:200; *v:v*). Following the primary antibody incubation, slides were washed and incubated with a secondary antibody anti-rabbit antibody conjugated with Alexa Fluor488 (Molecular Probes) diluted 1:250 (*v:v*) for IZUMO1 and 1:500 for GSTM3 in blocking solution. Finally, a drop of 10 μL of Vectashield mounting medium containing DAPI was added, and a coverslip was placed prior to sealing with nail varnish.

All samples were evaluated under a confocal laser-scanning microscope (CLSM, Nikon A1R; Nikon Corp., Tokyo, Japan). In negative controls, the primary antibodies were omitted. Furthermore, the specificity of the primary antibodies was confirmed by separate peptide competition assays. Samples were incubated with GSTM3- (ref. AAP53561; Aviva Systems Biology) and IZUMO1-specific (ref. NBP1-83086PEP; Novus Biologicals) blocking peptides, which were 10 times in excess with regard to the corresponding primary antibody (see Additional file 3). The immunofluorescence protocol is described in detail in Additional file 1.

### Statistical analysis

Data were analysed with a statistical package (IBM SPSS for Windows 25.0; Armonk, NY, USA). First, normality and homogeneity of variances were checked with Shapiro-Wilk and Levene tests, respectively. When needed, data were transformed with arcsin √x and re-checked for normality and homogeneity of variances. Each biological replicate was considered as a statistical case.

Groups of ejaculates with good (GFE) and poor (PFE) freezability were set on the basis of their total and progressive sperm motilities, and sperm viability (% SYBR14+/PI- spermatozoa) at 30 min through a two-step hierarchical cluster analysis using the log-likelihood as a distance measure and the Bayesian Schwarz criterion to build the groups. After establishing the two groups of ejaculates (GFE and PFE), sperm function parameters (i.e. % Viable spermatozoa with low lipid disorder, % Viable spermatozoa with low superoxide levels…) and relative content of IZUMO1 and GSTM3 were compared between these two groups and before and after cryopreservation (pre-frozen, FT 30 min, FT 240 min) with a linear mixed model (repeated measures). In this model, the cryopreservation step was the within-subjects factor, the ejaculate group (GFE vs. PFE) was the between-subjects factor and the boar was the random-effects factor. Post-hoc Sidak test was used for pair-wise comparisons. Finally, Pearson correlation coefficients were calculated between relative content of IZUMO1 and GSTM3 in pre-frozen and frozen-thawed sperm and all quality parameters evaluated in pre-frozen and 30- and 240-min post-thaw sperm. Data are shown as mean ± SEM. For all analyses, the level of significance was set at *P* ≤ 0.05.

## Results

### Classification of boar ejaculates in GFE and PFE groups

Sperm viability assessed at 30 min post-thaw was used to classify ejaculates as GFE and PFE. Although no differences were found between groups in pre-frozen samples (*P* > 0.05), sperm total and progressive motility, and viability were higher (*P* < 0.05) in GFE than in PFE at both 30 and 240 min post-thaw (Table 1).

**Table 1:** Sperm quality parameters in pre-frozen (P-F) and frozen–thawed (F-T) sperm, 30 (F-T-30 min) and 240 (F-T-240 min) min after thawing (mean ± SEM)

Parameter	Classification	P-F	F-T-30 min	F-T-240 min
% Total motile spermatozoa	GFE	72.52 ± 4.49^a,1^	30.10 ± 1.91^a,2^	8.14 ± 1.29^a,3^
	PFE	71.45 ± 6.53^a,1^	4.45 ± 1.47^b,2^	1.97 ± 0.34^b,2^
% Progressive motile spermatozoa	GFE	54.25 ± 2.60^a,1^	21.04 ± 1.77^a,2^	5.02 ± 1.48^a,3^
	PFE	50.09 ± 5.01^a,1^	1.83 ± 0.99^b,2^	0.28 ± 0.09^b,2^
% SYBR-14^+^ /PI^−^ spermatozoa	GFE	81.20 ± 1.65^a,1^	28.77 ± 2.85^a,2^	21.56 ± 3.46^a,3^
	PFE	79.84 ± 3.42^a,1^	5.60 ± 1.56^b,2^	3.79 ± 1.01^b,2^
% M540^−^/YO-PRO-1^−^ spermatozoa	GFE	75.13 ± 3.23^a,1^	24.36 ± 2.33^a,2^	14.59 ± 2.49^a,3^
	PFE	73.32 ± 3.58^a,1^	6.26 ± 1.51^b,2^	2.72 ± 0.72^b,2^
% JC1_agg_ spermatozoa	GFE	79.14 ± 3.04^a,1^	34.29 ± 2.36^a,2^	27.57 ± 5.51^a,2^
	PFE	77.50 ± 3.28^a,1^	16.50 ± 3.28^b,2^	12.75 ± 5.88^a,2^
% E^−^/YO-PRO-1^−^ spermatozoa	GFE	80.01 ± 2.23^a,1^	21.92 ± 2.26^a,2^	14.09 ± 3.15^a,3^
	PFE	76.10 ± 2.79^a,1^	8.86 ± 1.10^b,2^	3.39 ± 1.82^b,2^

Each ejaculate was classified as having good (GFE) or poor freezability (PFE). Different superscript numbers (^1,2,3^) indicate significant differences (*P* < 0.05) between conditions (pre-frozen (P-F), F-T-30 min and F-T-240 min). Different superscript letters (^a,b^) indicate significant differences(*P* < 0.05) between GFE and PFE in a given parameter

### Effects of cryopreservation on sperm quality parameters

Sperm quality parameters from boar ejaculates classified as GFE and PFE, were assessed before and after cryopreservation and are summarized in Table 1. Regarding viable and total and progressive motile spermatozoa, no differences were found in pre-frozen samples between GFE and PFE (*P* > 0.05). Although motility and viability were decreased after cryopreservation in both GFE and PFE groups, this decrease was more dramatic in PFE than in GFE, at both 30 and 240 min after thawing (*P* < 0.05).

Concerning the evaluation of sperm membrane lipid disorder (M540^−^/YO-PRO-1^−^), PFE showed higher membrane lipid disorder than GFE at 30- and 240-min after thawing (*P* < 0.05). As expected, the percentage of viable spermatozoa with low membrane lipid disorder were lower 30 and 240 min post-thaw than samples before freezing in both GFE and PFE (*P* < 0.05).

Regarding mitochondrial membrane potential, GFE contained a higher percentage of sperm with high mitochondrial membrane potential (JC-1_agg_), than PFE at 30 min post-thaw (*P* < 0.05). However, 240 min after thawing of the samples, no differences could be observed between groups. Finally, intracellular levels of superoxides (O_2_^−^▪) were higher in PFE than in GFE (*P* < 0.05). Moreover, a decrease of viable spermatozoa with high superoxide levels was observed after freeze-thawing in both GFE and PFE (*P* < 0.05).

### Effects of cryopreservation on the localisation of IZUMO1 and GSTM3

Localisation of IZUMO1 and GSTM3 was determined in pre-frozen and frozen-thawed boar spermatozoa by immunofluorescence. No differences were found in IZUMO1- and GSTM3-localisation patterns between GFE and PFE groups.

**Fig. 1:**
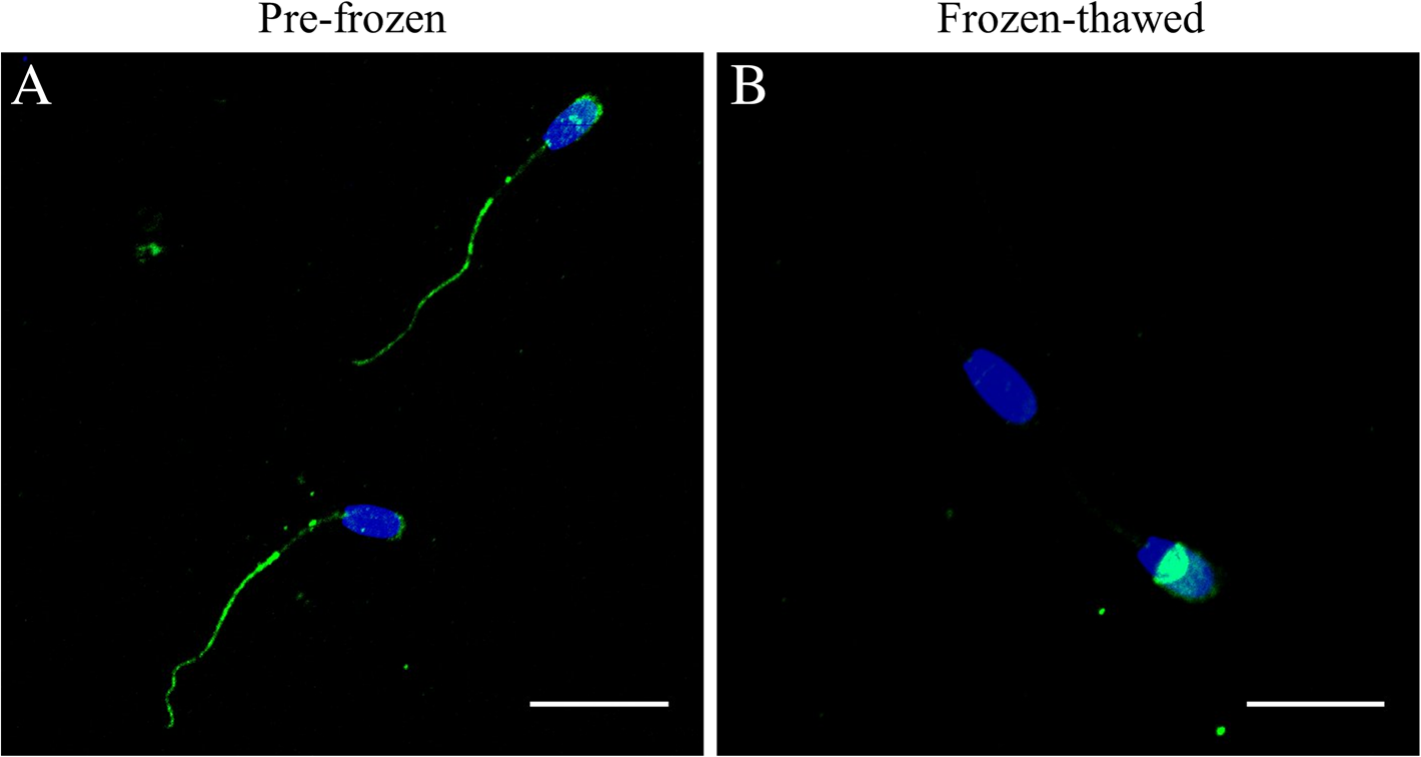
Immunolocalisation of IZUMO1 in (**a**) pre-frozen and (**b**) frozen-thawed boar spermatozoa. Nucleus is shown in blue (DAPI), whereas IZUMO1 is shown in green (FITC). Scale bars: **A**: 20 μm; **B**: 14 μm

Figure 1 shows the representative localisation patterns of IZUMO1 before and after cryopreservation of boar sperm. Two IZUMO1 localisation patterns were found in pre-frozen boar sperm: 1) fluorescence signal was located in the principal and end pieces of the tail of all spermatozoa, whereas 2) only some cells showed an IZUMO1-signal in the acrosome. With regard to frozen-thawed samples, IZUMO1 was exclusively localised in the equatorial segment. Interestingly, some pre-frozen and frozen-thawed sperm did not exhibit a positive IZUMO1-signal.

**Fig. 2:**
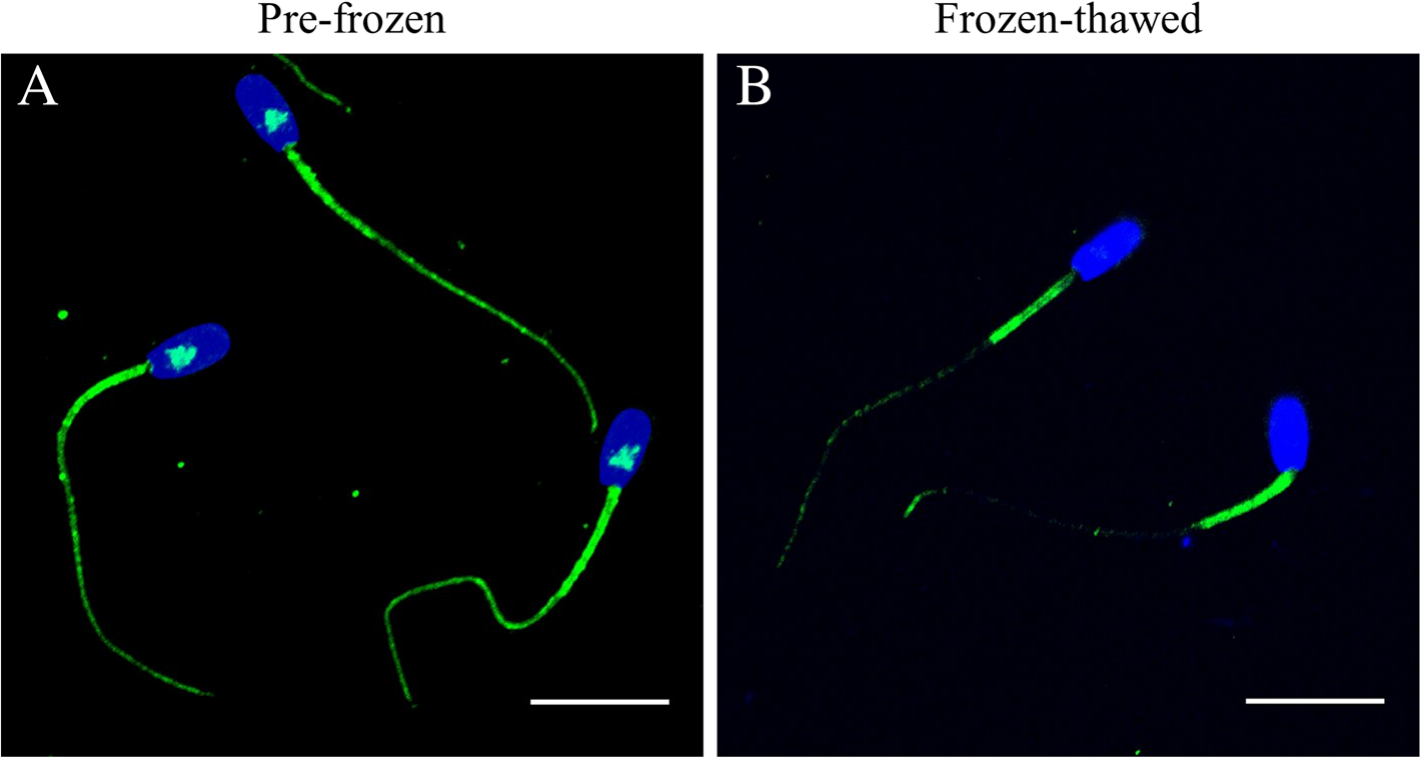
Immunolocalisation of GSTM3 in (**a**) pre-frozen and (**b**) frozen-thawed boar spermatozoa. Nucleus is shown in blue (DAPI), whereas GSTM3 is shown in green (FITC). Scale bars: **A-B**: 14.1 μm

Figure 2 shows representative localisation patters of GSTM3 in pre-frozen and frozen-thawed boar sperm. Sperm GSTM3 was located in the equatorial subdomain of the head and in mid-, principal and end-pieces of the tail before cryopreservation. Frozen-thawed boar sperm showed an intense GSTM3 signal in the mid-piece area only, being absent from the principal and end pieces of the tail and from the equatorial subdomain of the head. As opposed to the IZUMO-1 stained samples, all spermatozoa showed GSTM3 fluorescence signal.

### Relative abundances of IZUMO1 and GSTM3 during cryopreservation

**Fig. 3:**
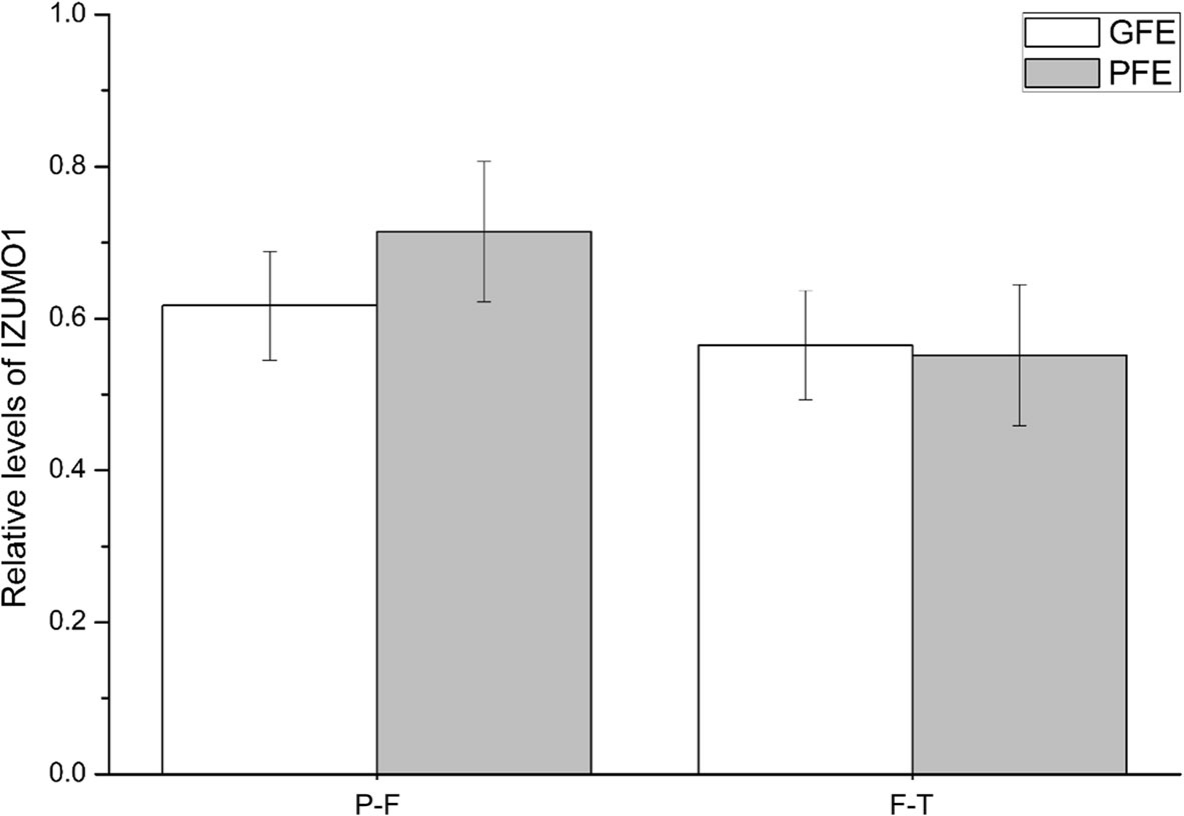
Relative abundances of IZUMO1 as mean ± standard error of the mean (SEM) of GFE and PFE in pre-frozen (P-F) and frozen-thawed (F-T) boar spermatozoa. Values were normalized using α-tubulin protein as an internal standard. Each sperm sample were evaluated three times

Western blot analysis of IZUMO1 evidenced a single-band of ~ 48 kDa band in both pre-frozen and frozen-thawed boar sperm (see Additional file 4). Stripping of membranes and incubation with α-tubulin was performed in order to confirm the same amount of total protein was loaded in all samples. The results showed a band at ~ 50 kDa in every sample. Following quantifications of relative IZUMO1-conent, no differences were observed between groups (GFE vs. PFE) either before or after cryopreservation (Fig. 3).

**Fig. 4:**
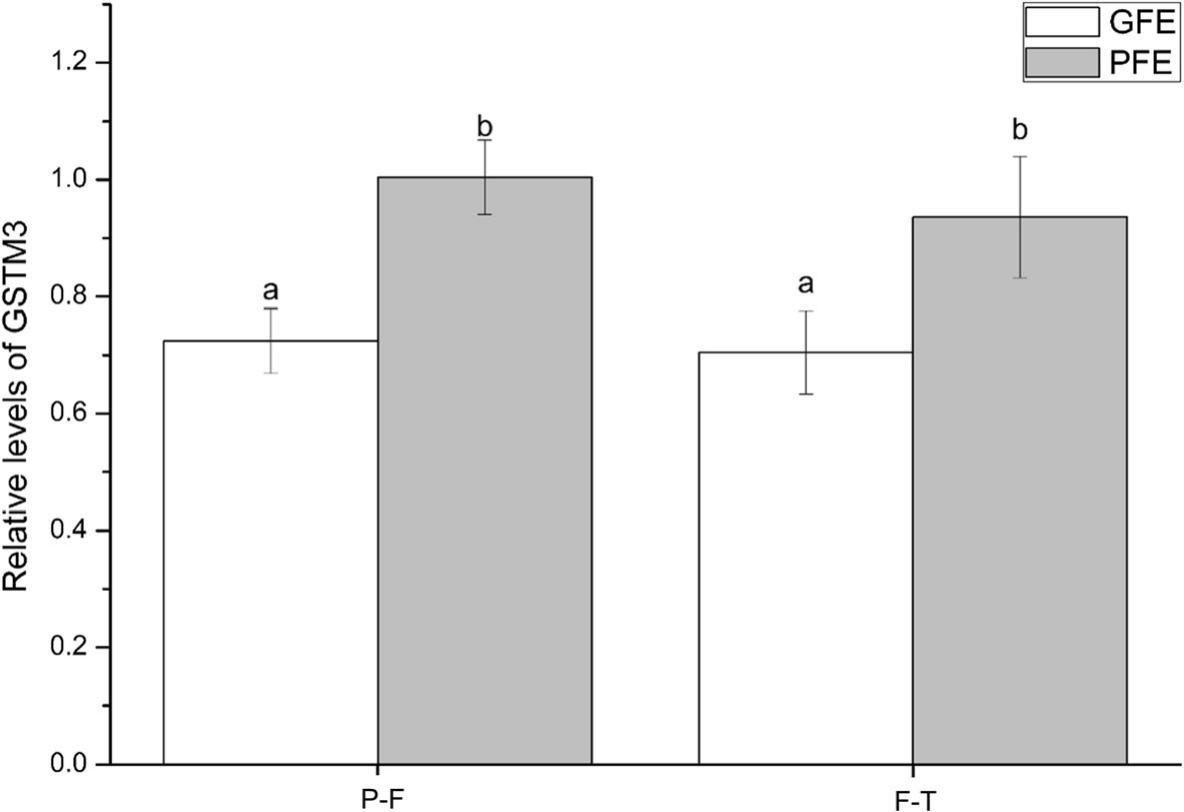
Relative abundances of GSTM3 as mean ± standard error of the mean (SEM) of GFE and PFE in pre-frozen (P-F) and frozen-thawed (F-T) boar spermatozoa. Values were normalized using α-tubulin protein as an internal standard. Each sperm sample were evaluated two times

Immunoblotting of GSTM3 showed a single-band pattern of ~ 25 kDa in pre-frozen and frozen-thawed boar sperm (see Additional file 4). Weak-intensity bands at ~ 28 and ~ 48 kDa were also observed. The incubation with α-tubulin showed a ~ 50 kDa band in all samples. Quantification of relative levels of GSTM3 evidenced differences in protein content between GFE and PFE in both pre-frozen (0.72 INT·mm^2^ ± 0.15 INT·mm^2^ vs. 1.00 INT·mm^2^ ± 0.14 INT·mm^2^; *P* < 0.05) and frozen-thawed (0.70 INT·mm^2^ ± 0.19 INT·mm^2^ vs. 0.96 INT·mm^2^ ± 0.20 INT·mm^2^; *P* < 0.05) samples, with PFE showing higher relative GSTM3-levels than the GFE (Fig. 4).

### Correlations between relative contents of IZUMO1 and GSTM3 and sperm quality parameters

Tables 2 and 3 show Pearson correlation coefficients between relative contents of IZUMO1 and GSTM3, respectively, and sperm quality parameters in pre-frozen and frozen-thawed sperm (30 and 240 min post-thaw). No correlation between the relative content of these proteins and pre-frozen sperm quality parameters was found, nor between relative IZUMO1-abundance and post-thaw sperm quality parameters. However, relative levels of pre-frozen GSTM3 were negatively correlated with percentages of viable spermatozoa (SYBR14^+^/PI^−^), viable spermatozoa with low membrane lipid disorder (M540^−^/YO-PRO-1^−^), viable spermatozoa with low levels of superoxides (E^−^/YO-PRO-1^−^), and spermatozoa with high mitochondrial membrane potential (JC1_agg_) 30 min post-thaw (*P* < 0.05). In addition, relative levels of GSTM3 after cryopreservation were negatively correlated (*P* < 0.05) with percentages of viable spermatozoa with low levels of superoxides (E^−^/YO-PRO-1^−^) and high mitochondrial membrane potential (JC1_agg_) 30 min post-thaw. Finally, percentages of viable spermatozoa (SYBR14^+^/PI^−^) and viable spermatozoa with low membrane lipid disorder (M540^−^/YO-PRO-1^−^) at 240 min post-thaw were negatively correlated (*P* < 0.05) with the relative levels of GSTM3 evaluated in pre-frozen and frozen-thawed sperm.

**Table 2:** Correlation coefficients between relative IZUMO1 abundances in both pre-frozen (P-F) and frozen thawed (F-T) spermatozoa and sperm quality parameters in pre-frozen (P-F) and frozen-thawed spermatozoa, evaluated 30 (F-T-30) and 240 (F-T-240) min post-thaw

		Relative levels of P-F IZUMO1	Relative levels of F-T IZUMO1
P-F	% Progressive motile spermatozoa	0.37	0.19
	% Total motile spermatozoa	0.12	0.38
	% SYBR-14^+^/PI^−^ spermatozoa	0.24	− 0.23
	% M540^−^/YO-PRO-1^−^ spermatozoa	0.21	0.21
	% JC1_agg_ spermatozoa	−0.27	− 0.51
	% E^−^/YO-PRO-1^−^ spermatozoa	0.22	− 0.55
F-T-30	% Progressive motile spermatozoa	0.26	−0.25
	% Total motile spermatozoa	0.23	−0.27
	% SYBR-14^+^/PI^−^ spermatozoa	−0.05	−0.08
	% M540^−^/YO-PRO-1^−^ spermatozoa	−0.05	0.03
	% JC1_agg_ spermatozoa	−0.11	−0.01
	% E^−^/YO-PRO-1^−^ spermatozoa	−0.25	− 0.03
F-T-240	% Progressive motile spermatozoa	0.25	−0.12
	% Total motile spermatozoa	0.20	−0.11
	% SYBR-14^+^/PI^−^ spermatozoa	−0.05	0.08
	% M540^−^/YO-PRO-1^−^ spermatozoa	−0.15	0.11
	% JC1_agg_ spermatozoa	−0.31	−0.41
	% E^−^ / YO-PRO-1^−^ spermatozoa	0.02	0.23

**Table 3:** Correlation coefficients between relative GSTM3-abundances in both pre-frozen (P-F) and frozen thawed (F-T) spermatozoa and sperm quality parameters in pre-frozen (P-F) and frozen-thawed spermatozoa, evaluated 30 (F-T-30) and 240 (F-T-240) min post-thaw. *P < 0.05; **P < 0.01

		Relative levels of P-F GSTM3	Relative levels of F-T GSTM3
P-F	% Progressive motile spermatozoa	0.29	0.40
	% Total motile spermatozoa	0.21	0.31
	% SYBR-14^+^/PI^−^ spermatozoa	−0.23	−0.24
	% M540^−^/YO-PRO-1^−^ spermatozoa	− 0.11	−0.03
	% JC1_agg_ spermatozoa	0.06	0.09
	% E^−^/YO-PRO-1^−^ spermatozoa	−0.48	−0.27
F-T-30	% Progressive motile spermatozoa	−0.04	0.11
	% Total motile spermatozoa	−0.08	0.05
	% SYBR-14^+^/PI^−^ spermatozoa	−0.67*	−0.50
	% M540^−^/YO-PRO-1^−^ spermatozoa	−0.70*	− 0.54
	% JC1_agg_ spermatozoa	−0.80**	−0.70**
	% E^−^/YO-PRO-1^−^ spermatozoa	−0.74**	− 0.71**
F-T-240	% Progressive motile spermatozoa	0.08	0.19
	% Total motile spermatozoa	−0.08	0.04
	% SYBR-14^+^/PI^−^ spermatozoa	−0.75**	−0.64*
	% M540^−^/YO-PRO-1^−^ spermatozoa	−0.75**	− 0.71**
	% JC1_agg_ spermatozoa	−0.56	0.56
	% E^−^ / YO-PRO-1^−^ spermatozoa	−0.45	−0.40

## Discussion

Alterations in the levels of membrane-bound fertility-related proteins, such as IZUMO1 and GSTM3, have been associated to male subfertility [8]. Moreover, cryopreservation is known to alter the content and localisation of several sperm proteins [2]. Based on these facts, the current work aimed to evaluate the localisation of IZUMO1 and GSTM3 and quantify their relative levels in boar spermatozoa before and after cryopreservation, comparing ejaculates with good (GFE) and poor (PFE) freezability. The results reported in this work show that: 1) both IZUMO1 and GSTM3 undergo relocation due to cryopreservation; however, 2) their relative abundance levels are not altered by this process; 3) remarkably, relative GSTM3-content in pre-frozen sperm is correlated with post-thaw sperm quality, and is higher in PFE than in GFE.

The presence of IZUMO1 in boar sperm was assessed by immunoblotting, and a single-band of ~ 48 kDa was found in both pre-frozen and frozen-thawed boar sperm. Although the predicted molecular mass for IZUMO1 is ~ 37 kDa, our results are in agreement with those reported in boar [17] and bull [16] spermatozoa. This data suggests that IZUMO1 could undergo post-translation modifications (such as glycosylation or phosphorylation) in both species during sperm maturation, and that these modifications increase its predicted molecular weight.

Regarding protein localisation, IZUMO1 was found in the acrosome and principal and end tail pieces of boar spermatozoa before freezing. Translocation from this location to the equatorial segment was observed in frozen-thawed sperm. This relocation is in agreement with the results previously reported by Fukuda, et al. [16], who found IZUMO1 to be located in the whole equatorial segment of frozen-thawed bull sperm. Moreover, a similar relocation has been reported in mouse sperm during acrosome reaction. Mice IZUMO1 relocates from both the inner and outer acrosome membranes to the equatorial segment due to the acrosome reaction [30]. It is worth noting that IZUMO1-staining was unexpectedly found in the sperm tail of pre-frozen, but not frozen-thawed, boar sperm. That being said, no differences in the localisation of this protein between GFE and PFE were observed, either before and after cryopreservation.

Our results demonstrate that IZUMO1 content was similar in pre-frozen and frozen-thawed sperm, without differences being observed between GFE and PFE. Our finding contrasts with those reported by Fukuda et al. [16], who showed a reduction of IZUMO1 in cryopreserved bull samples compared with pre-frozen sperm due to the loss of acrosome integrity during this procedure. However, it should be noted that Fukuda et al. [16] assessed this reduction of protein content through immunofluorescence analysis, whereas in this study, quantification of Western blot bands was carried out. In fact, similarly to what it is being reported in this study on boar sperm, relocation (rather than complete loss) of IZUMO1 occurs during capacitation and acrosome reaction in mouse sperm [31].

Western blot analysis showed a single band pattern of ~ 25 kDa in pre-frozen and frozen-thawed boar sperm when membranes were probed with an anti- GSTM3 antibody. Similarly, Kwon et al. [21] also reported a single band of ~ 27 kDa for GSTM3, the predicted molecular mass of boar GSTM3 being 26.6 kDa. Additionally, we also found an additional weak band of ~ 28 kDa in GSTM3 membranes that was not observed in the peptide competition assay. These slight differences in molecular weight could be again due to post-translational modifications of GSTM3. Moreover, another low-intensity band of ~ 48 kDa, which also disappeared in peptide competition assay, was present on GSTM3 membranes. The presence of that band may be due to the homodimerisation of this protein [32].

Since immunoblotting analysis did not show any loss of GSTM3 during cryopreservation, a relocation rather than a loss of this protein appears to occur due the freeze-thawing procedures. This was confirmed by immunofluorescence, where we observed that GSTM3 underwent relocation from the equatorial subdomain of the head, and mid-, principal and end pieces of the tail to the mid-piece during cryopreservation. In addition, no differences were observed between sperm from GFE and PFE in GSTM3-localisation. Previous studies in goat and human sperm [13, 14, 19] found GSTM3 at the apical region of the acrosome. These works also reported that this protein translocates to the equatorial and posterior acrosome regions during capacitation, and is lost from the acrosome upon acrosome reaction. On the other hand, Kumar et al. [22] showed that buffalo GSTM3 is localised over the connecting, mid-, principal, and end pieces of the tail in sperm before cryopreservation. These authors also demonstrated that after cryopreservation, GSTM3 migrated to the mid-piece. Remarkably, while this localisation pattern in pre-frozen and post-thawed buffalo sperm was similar to that observed in our study, it differed from that found in goat and human sperm.

Regarding GSTM3 content during cryopreservation, the results of the present study differ from those reported by Kumar et al. [22] in buffalo sperm. While these authors found a decrease in GSTM3 content after cryopreservation, we did not observe this effect. These differences might arise from the differences in techniques used between this study and the study of Kumar et al. [22]. Perhaps the most interesting result of our study, however, was the difference in relative GSTM3 content found between GFE and PFE, in both pre-frozen and post-thawed sperm. Ejaculates classified as PFE showed higher levels of GSTM3 than GFE. Taking into account that GSTM3 is involved in cell protection against oxidative stress [20], and PFE exhibit lower post-thaw sperm quality, it is reasonable to suggest that the higher levels of this protein in PFE could represent a mechanism to reduce oxidative stress.

The present study also attempted to find a relationship between sperm quality parameters and the relative amounts of IZUMO1 and GSTM3. Interestingly, a negative correlation between sperm quality parameters at 30 min post-thaw and relative levels of GSTM3 in pre-frozen spermatozoa was observed. Higher GSTM3-content before cryopreservation was related to a lower percentage of viable spermatozoa (SYBR14^+^/PI^−^), viable spermatozoa with low membrane disorder, viable spermatozoa with low levels of intracellular superoxide levels, and spermatozoa with low mitochondrial membrane potential at post-thaw. Interestingly, other studies have demonstrated that an overexpression of GSTM3 in pre-frozen sperm is related with small litter sizes in boars [21] and with lower sperm quality in humans [33]. In addition, Hemachand and Shaha [20] reported that membrane-bound GSTMs eliminate ROS via extracellular glutathione and, consequently, prevent lipid membrane peroxidation, a process highly damaging to sperm membrane integrity [34]. This protection against oxidative stress exerted by this protein could preserve sperm motility, viability, mitochondrial status, oocyte binding capacity and fertilising ability. In fact, in the present study, a decrease of mitochondrial activity, and an increase in superoxide production and lipid disorder after cryopreservation were observed in both GFE and PFE. Thus, the relocation of GSTM3 to the mid-piece in response to cryopreservation reported in this study could be a mechanism of sperm to reduce oxidative stress during freeze-thawing.

Collectively, our results indicate that higher relative content of GSTM3 in pre-frozen sperm is related to lower sperm cryotolerance, and could be related to the fertility-associated issues of frozen-thawed sperm. While our findings demonstrate the reliability of GSTM3 as a sperm cryotolerance marker, further research including *in vitro* and *in vivo* fertilisation experiments is required to elucidate whether this protein is also a marker of their fertilising ability in both pre-frozen and frozen-thawed boar semen.

## Conclusion

On the basis of immunofluorescence analysis and in accordance with studies in other species, relocation of IZUMO1 and GSTM3 occurs in response to cryopreservation. On the other hand, Western blot analysis shows no significant variations of IZUMO1 and GSTM3 content along the cryopreservation protocol. Nevertheless, although the content of IZUMO1 in pre-frozen boar sperm was found not to be related to their cryotolerance, sperm GSTM3 content before cryopreservation was higher in PFE than in GFE. These data indicate that GSTM3 could be used as a freezability marker in boar sperm. Finally, since no significant reduction of IZUMO1 and GSTM3 content has been reported during cryopreservation procedures, it is reasonable to suggest that the impaired fertilising ability of cryopreserved boar spermatozoa could be partially related to the abnormal translocation of both fertility-related proteins. However, additional *in vitro* and *in vivo* fertilisation essays are required to confirm this hypothesis.

## Additional files

Additional file 1: Supplementary information for Materials and Methods. (DOC 53 kb)

Additional file 2: Western blots resulting from incubation with the (**A**) IZUMO1-antibody together with the IZUMO1-blocking peptide (IZUMO1 – blocking peptide) and its loading control (α-tubulin); and (**B**) GSTM3-antibody with GSTM3-blocking peptide (GSTM3 – blocking peptide) and its loading control (α-tubulin). Lanes P-F: pre-frozen sperm. Lanes F-T: frozen-thawed sperm. Lanes GFE: good freezability ejaculates. Lanes PFE: poor freezability ejaculates. (TIF 1214 kb)

Additional file 3: Immunofluorescence of (**A**) IZUMO1 negative control; (**B**) IZUMO1-antibody incubation with the IZUMO1-blocking peptide; (**C**) GSTM3 negative control and (**D**) GSTM3-antibody incubation with the GSTM3-blocking peptide. Nucleus is shown in blue (DAPI). Scale bars: A-B: 18 μm; C-D: 14 μm. (TIF 514 kb)

Additional file 4: Representative Western blot resulting from incubation with the (**A**) IZUMO1 antibody and its loading control (α-tubulin) and (**B**) GSTM3 antibody and its loading control (α-tubulin). Lanes P-F: pre-frozen sperm. Lanes F-T: frozen-thawed sperm. Lanes GFE: “good” freezability ejaculates. Lanes PFE: “poor” freezability ejaculates. (TIF 1479 kb)

## Data Availability

The datasets used and/or analysed during the current study are available from the corresponding author on reasonable request.

## Abbreviations

AI: Artificial insemination
ALH: Amplitude of lateral head displacement
ART: Assisted reproduction techniques
BCF: Beat cross frequency
GFE: Good freezability ejaculates
HE: Hydroethidine
IgSF: Immunoglobulin superfamily
ISAC: International Society for Advancement of Cytometry
IVF: *in vitro* fertilisation
JC-1: 5,5′,6,6′-tetrachloro-1,1′,3,3’tetraethyl-benzimidazolylcarbocyanine iodide
LIN: Linearity
M540: Merocyanine 540
PFE: Poor freezability ejaculates
PI: Propidium iodide
PMOT: Progressive motility
ROS: Reactive oxygen species
SEM: Standard error of the mean
STR: Straightness
VAP: Average path velocity
VCL: Curvilinear velocity
VSL: Straight line velocity
