# Supplementary material for: GSTM3, but not IZUMO1, is a cryotolerance marker of boar sperm
Source: J Anim Sci Biotechnol. 2019 Aug 5;10:61. doi: 10.1186/s40104-019-0370-5 (PMC6681495; doi:10.1186/s40104-019-0370-5)
Supplement: Supplementary file 1 — Supplementary information for Materials and Methods. (DOC 53 kb) [file 40104_2019_370_MOESM1_ESM.doc]

**Supplementary information for Materials and Methods**

**Flow cytometry analyses**

*General information*

Four sperm parameters (plasma membrane integrity, sperm membrane lipid disorder, mitochondrial membrane potential and intracellular levels of superoxides [O_2_^-^●]) were evaluated. All sperm samples were diluted with phosphate buffered saline (PBS) 1× to a final concentration of 5×10^6^ cells per mL in a final volume of 0.6 mL before they were stained with the corresponding protocol. The flow cytometry assessments were conducted using a Cell Laboratory QuantaSC cytometer (Beckman Coulter; Fullerton, CA, USA), and samples were excited with an argon ion laser (488 nm) set at a power of 22 mW. The cytometer provided the electronic volume (EV) and the side scatter (SS) for each event. Three optical filters (FL-1, FL-2 and FL-3) were used. FL-1 served to detect green fluorescence (SYBR14, YO-PRO-1 and 5,5’,6,6’-tetrachloro-1,1’,3,3’tetraethyl-benzimidazolylcarbocyanine iodide monomers (JC-1 monomers; JC-1_mon_)), FL-2 was used to detect orange fluorescence (JC-1 aggregates; JC-1_agg_) and FL-3 allowed to detect red fluorescence (merocyanine 540 (M540), hydroethidine (HE) and propidium iodide (PI)). Signals were logarithmically amplified, and photomultiplier settings were adjusted to particular staining methods. A total of three technical replicates, with a minimum of 10,000 events per replicate, were evaluated for each ejaculate and sperm parameter.

Flowing Software (Ver. 2.5.1; University of Turku, Finland) was used to perform flow cytometric data analysis, following the recommendations of the International Society for Advancement of Cytometry (ISAC). The corresponding mean ± standard error of the mean (SEM) was subsequently calculated.

*Plasma membrane integrity*

Viability of pre-frozen and frozen-thawed sperm was evaluated by assessing their membrane integrity using the LIVE/DEAD sperm viability kit (Molecular Probes, Eugene, OR, USA), which follows the protocol of Garner and Johnson [1]. Briefly, spermatozoa were stained with SYBR14 (final concentration: 100 nmol/L) for 10 min at 38ºC in the dark and with PI (final concentration: 12 µmol/L) for 5 min at the same conditions. Combination of SYBR14 and PI resulted in three sperm populations in flow cytometry dot-plots: (i) viable green-stained spermatozoa (SYBR-14^+^/PI^-^); (ii) non-viable red-stained spermatozoa (SYBR14^-^/PI^+^); and (iii) moribund spermatozoa stained both green and red (SYBR14^+^/PI^+^). Non-sperm particles (debris; SYBR14^-^/PI^-^) appeared in the left-bottom quadrant. Viable green-stained spermatozoa were used to assess sperm viability. SYBR-14 spill over into the FL3-channel was compensated (2.45%).

*Sperm membrane lipid disorder*

Membrane lipid disorder of pre-frozen and frozen-thawed sperm was evaluated by M540 and YO-PRO-1 co-staining, following the procedure of Rathi et al. [2] with minor modifications by Yeste et al. 2014 [3]. Briefly, spermatozoa were incubated with M540 (final concentration: 2.6 µmol/L) and YO-PRO-1 (final concentration: 25 nmol/L) for 10 min at 38ºC in the dark. Red fluorescence from M540 was collected through FL-3 and green fluorescence from YO-PRO-1 was collected through FL-1. The combination of both fluorochromes resulted in four populations: (i) viable spermatozoa with low membrane lipid disorder (M540^-^/YO-PRO-1^-^); (ii) viable spermatozoa with high membrane lipid disorder (M540^+^/YO-PRO-1^-^); (iii) non-viable spermatozoa with low membrane lipid disorder (M540^-^/YO-PRO-1^+^) and (iv) non-viable spermatozoa with high membrane lipid disorder (M540^+^/YO-PRO-1^+^). Data were not compensated. The percentage of viable spermatozoa with low membrane lipid disorder (M540^-^/YO-PRO-1^-^) was corrected using the debris particles found in SYBR14/PI staining. The percentages of the other three populations were also recalculated.

*Mitochondrial membrane potential*

Mitochondrial membrane potential of pre-frozen and frozen-thawed sperm was evaluated following a protocol modified from Ortega-Ferrusola et al. [4]. All samples were incubated with JC-1 (final concentration: 0.3 µmol/L) for 30 min at 38ºC in the dark. High mitochondrial potential causes JC-1 aggregates that emit orange fluorescence collected through FL-2, whereas low mitochondrial potential causes JC-1 monomers that emit green fluorescence collected through FL-1. Consequently, a total of three sperm populations were observed in flow cytometry dot-plots: (i) spermatozoa with low mitochondrial membrane potential (green-stained); (ii) spermatozoa with high mitochondrial membrane potential (orange-stained) and (iii) spermatozoa with heterogeneous mitochondria (green and orange-stained in the same cell). Debris particles found in SYBR14/PI staining were removed from the JC-1 double-negative population and all the other percentages were recalculated. Spermatozoa considered having high mitochondrial membrane potential (JC1_agg_), resulted from the sum of (ii) and (iii) populations.

*Intracellular ROS levels: O_2_^-^●*

Sperm oxidative stress was evaluated by assessing intracellular levels of hydrogen superoxides (O_2_^-^●), following a modification of the procedure described by Guthrie and Welch [5]. In brief, sperm samples were incubated with HE (final concentration of 4 µM) and with YO-PRO-1 (final concentration of 40 nmol/L), at 38°C and for 20 min in the dark. The oxidation of HE to ethidium (E^+^) was detected as red fluorescence through FL-3 and green fluorescence from YO-PRO-1 was detected through FL-1. Combination of these fluorochromes resulted in four populations: (i) non-viable spermatozoa with high superoxide levels (E^+^ YO-PRO-1^+^), (ii) viable spermatozoa with high superoxide levels (E^+^/YO-PRO-1^-^), (iii) non-viable spermatozoa with low superoxide levels (E^-^/YO-PRO-1^+^); and (iv) viable spermatozoa with low superoxide levels cells (E^-^/YO-PRO-1^-^). The percentage of viable spermatozoa with low superoxide levels (E^-^/YO-PRO-1^-^) was corrected using the debris particles found in SYBR14/PI staining. The percentages of the other three populations were also recalculated. YO-PRO-1 spill over into the FL3-channel was compensated (5.06%).

**Western blot analysis**

Pre-frozen and frozen-thawed boar sperm were used for Western blot analysis. For pre-freezing conditions, a volume of 15 mL was centrifuged at 1,500 × g at 17ºC for 5 min. With regard to frozen-thawed boar sperm, 3.5 mL aliquots were centrifuged twice at 3,000 × g at 17ºC for 5 min. Following this, an additional centrifugation step at 1,500 × g at 17ºC for 5 min was performed prior to storing the sperm pellets at -80ºC until protein extraction.

*Protein extraction*

All pellet samples were resuspended in 400 µL of lysis buffer (8 mol/L urea [BioRad, Hercules, CA, USA], Tris-HCl 50 mmol/L [BioRad] adjusted to pH 8.8, 1 % (v/v) Triton X-100 [Sigma-Aldrich], 2% (w/v) sodium dodecyl sulphate [SDS; Serva, Heidelberg, Germany], 2 mmol/L dithiothreitol [Sigma-Aldrich], 0.5% (v/v) Tween-20 [Panreac, Castellar del Vallès, Barcelona], sodium orthovanadate 700 mmol/L [Sigma-Aldrich] boiled at 95 ºC, 1 mmol/L phenyl-methane-sulfonylfluoride [PMSF; Sigma-Aldrich], and 1:100 (v:v) protease inhibitor cocktail [Sigma-Aldrich], adjusted to pH = 7.4) and transferred to Eppendorf tubes, which were incubated in agitation at 4ºC for 30 min. After incubation, all samples were sonicated thrice with five pulses every 2 min and centrifuged at 10,000 × g at 4 ºC for 15 min. Finally, supernatants were transferred to siliconized Eppendorf tubes and stored at -80ºC prior to protein quantification. Quantification of total protein in all samples was carried out in triplicate by a detergent compatible (DC) method (BioRad).

*Gel electrophoresis (SDS-PAGE) and Western blot analysis*

Ten micrograms of total protein were resuspended in Laemmli reducer buffer 2× (170 mmol/L Tris [Serva], 21 % glycerol [Panreac], 4.3 % SDS [Serva], 5 % beta-mercaptoethanol [BioRad], and traces of bromophenol blue [Panreac]) and water up to a total volume of 30 µL for each sample. All samples were boiled at 96ºC before proteins were loaded onto the upper stacking gel (Tris-glycine SDS – 5 % Polyacrylamide) and separated across the lower resolving gel (Tris-glycine SDS – 12 % Polyacrylamide). Electrophoretic protein separation was carried out at 80 V for 90 min (IEF Cell Protean System, BioRad). At that point, separated proteins from the gel were transferred onto polyvinyl fluoride membranes (Immobilion-P; Millipore, Darmstadt, Germany) using Trans-Blot® Turbo™ (BioRad). Membranes were blocked in blocking solution (10 mmol/L Tris [Panreac], 150 mmol/L NaCl [labKem, Mataró, Spain], and 0.05 % Tween-20 [Panreac]; pH=7.3, and 5 % bovine serum albumin [Roche Diagnostics, S.L., Basel, Switzerland]) for 1 hour with agitation at room temperature. Blocked membranes were then incubated overnight with agitation at 4ºC with primary antibodies: anti-IZUMO1 polyclonal rabbit antibody (ref. NBP1–83086; Novus Biologicals, Littleton, CO, USA; 1:10,000; v:v) or anti-GSTM3 polyclonal rabbit antibody (ref. ARP53561_P050; Aviva Systems Biology, San Diego, USA; 1:20,000; v:v). Next, membranes were washed four times at room temperature with TBS 1×-Tween20 (10 mmol/L Tris [Panreac], 150 mmol/L NaCl [labKem], and 0.05 % Tween-20 [Panreac]; pH = 7.3) and incubated with secondary goat anti-rabbit antibody conjugated with horseradish peroxidase (HRP; Dako, Derkman A/S; Denmark) for an additional hour with agitation (1:15,000 (v:v) dilution for IZUMO1 and 1:25,000 (v:v) for GSTM3). Finally, membranes were washed three times with TBS 1×-Tween20 and bands were visualised with a chemiluminescent substrate (ImmobilionTM Western Detection Reagents, Millipore) and scanned with G:BOX Chemi XL 1.4 (SynGene, Frederick, MT, USA).

Following these steps, the membranes were stripped by incubation with agitation at room temperature with a stripping buffer (0.2 mol/L glycine [Serva], 0.1 % (w:v) SDS [Serva] and 1 % (v:v) Tween20 [Panreac]; pH adjusted at 2.2) two times for 10 min. Subsequently, membranes were washed with TBS 1× (10 mmol/L Tris [Panreac], and 150 mmol/L NaCl [labKem]; pH = 7.3) two times for 10 min, and then with TBS 1×-Tween20, two times for 5 min. Next, stripped membranes were blocked by incubating 1 h with agitation at room temperature in blocking solution, and then incubated overnight with agitation at 4ºC with anti-alpha-tubulin monoclonal mouse antibody (ref. MABT205, Millipore; 1:100,000, v:v). Thereafter, membranes were washed three times at room temperature for 5 min with TBS 1×-Tween20 and incubated with secondary anti-mouse HRP–conjugated polyclonal rabbit antibody (ref. P0260; Dako; 1:150,000, v:v) for 1 h with agitation at room temperature. Finally, membranes were washed three times at room temperature for 5 min with TBS 1×-Tween20, incubated with Immobilon Western Chemiluminescent HRP Substrate (Millipore) and scanned with G:BOX Chemi XL 1.4 (SynGene).

Three technical replicates per sample were evaluated and bands were quantified using Quantity One Version 4.6.2 software package (BioRad). The sum of pixel intensities inside a single volume minus the background volume was expressed as adjusted volume in density per square millimetre. Pattern quantifications were normalized using alpha-tubulin, and the corresponding mean ± SEM of each sample was subsequently calculated.

Peptide competition assays were performed in order to confirm the specificity of primary antibodies. With this purpose, immunising peptides for GSTM3 (ref. AAP53561; Aviva Systems Biology) and IZUMO1 (ref. NBP1-83086PEP; Novus Biologicals) were used together with primary antibodies in immunoblotting and were 20 times in excess with regard to their respective antibodies.

**Immunofluorescence**

Localisation of IZUMO1 and GSTM3 in pre-frozen and frozen-thawed boar sperm was evaluated through immunofluorescence. Sperm samples were diluted to a final concentration of 3×10^6^ cells per mL and then washed with PBS 1X at 155 × g and room temperature for 5 min. Washed sperm were fixed with 1.5% (w:v) paraformaldehyde at room temperature for 30 min. Then, samples were centrifuged twice at 155 × g at room temperature for 5 min and resuspended with PBS 1×. Two drops per sample were placed onto different slides, which had been previously rinsed with absolute ethanol. Subsequently, all slides were blocked and permeabilised with blocking solution consisting of TBS-Tween20 1X containing 0.25% (v:v) Triton X-100 and 3% (w:v) BSA. Then, all samples were incubated with primary antibodies anti-IZUMO1 polyclonal rabbit antibody (ref. NBP1–83086; Novus Biologicals, Littleton, CO, USA; 1:250; v:v) and anti-GSTM3 polyclonal rabbit antibody (ref. ARP53561_P050; Aviva Systems Biology; 1:200; v:v). This incubation was carried out overnight in a humid chamber in the dark at 4 ºC. In all cases, antibodies were diluted in 3% BSA in TBS-Tween20 1X with 0.25 % (v:v) Triton X-100. Following the primary antibody incubation, slides were washed five times with PBS 1× for 5 min. They were then incubated with a secondary antibody anti-rabbit antibody conjugated with Alexa Fluor488 (Molecular Probes) diluted 1:250 (v:v) for IZUMO1 and 1:500 for GSTM3 in blocking solution at room temperature for 1 h. Then, samples were washed five times with PBS 1× for 5 min. Finally, a drop of 10 µL of Vectashield mounting medium containing 125 ng per mL of 4,6-diamidino-2-phenylindole (DAPI; Vectorlabs, Burlingame, CA, USA) was added, and a coverslip was placed prior to sealing with nail varnish.

All samples were evaluated under a confocal laser-scanning microscope (CLSM, Nikon A1R; Nikon Corp., Tokyo, Japan). Samples were excited at 405 nm in order to localise the nuclei DAPI-stained nucleus, whereas excitation at 496 nm was used to determine the localisation of IZUMO1 and GSTM3. In negative controls, incubations with primary antibodies were omitted. Furthermore, the specificity of primary antibodies was confirmed by separate peptide competition assays. Samples were incubated with GSTM3- (ref. AAP53561; Aviva Systems Biology) and IZUMO1-specific (ref. NBP1-83086PEP; Novus Biologicals) blocking peptides, which were 10 times in excess with regard to the corresponding primary antibody.

**References cited in Supplementary Information for Materials and Methods**

1. Garner DL, Johnson LA. Viability assessment of mammalian sperm using SYBR-14 and propidium iodide. Biol Reprod. 1995;53:276–84.

2. Rathi R, Colenbrander B, Bevers MM, Gadella BM. Evaluation of in vitro capacitation of stallion spermatozoa. Biol Reprod. 2001;65:462–70.

3. Yeste M, Estrada E, Rivera del Álamo M-M, Bonet S, Rigau T, Rodríguez-Gil J-E. The increase in phosphorylation levels of serine residues of Ppotein HSP70 during holding time at 17°C is concomitant with a higher cryotolerance of boar spermatozoa. PLoS One. 2014;9:e90887.

4. Ortega-Ferrusola C, Sotillo-Galan Y, Varela-Fernandez E, Gallardo-Bolanos JM, Muriel A, Gonzalez-Fernandez L, et al. Detection of “apoptosis‐like” changes during the cryopreservation process in equine sperm. J Androl. 2007;29:213–21.

5. Guthrie HD, Welch GR. Determination of intracellular reactive oxygen species and high mitochondrial membrane potential in Percoll-treated viable boar sperm using fluorescence-activated flow cytometry. J Anim Sci. 2006;84:2089–100.
