# Supplementary figures and images for: GSTM3, but not IZUMO1, is a cryotolerance marker of boar sperm
Source: J Anim Sci Biotechnol. 2019 Aug 5;10:61. doi: 10.1186/s40104-019-0370-5 (PMC6681495; doi:10.1186/s40104-019-0370-5)

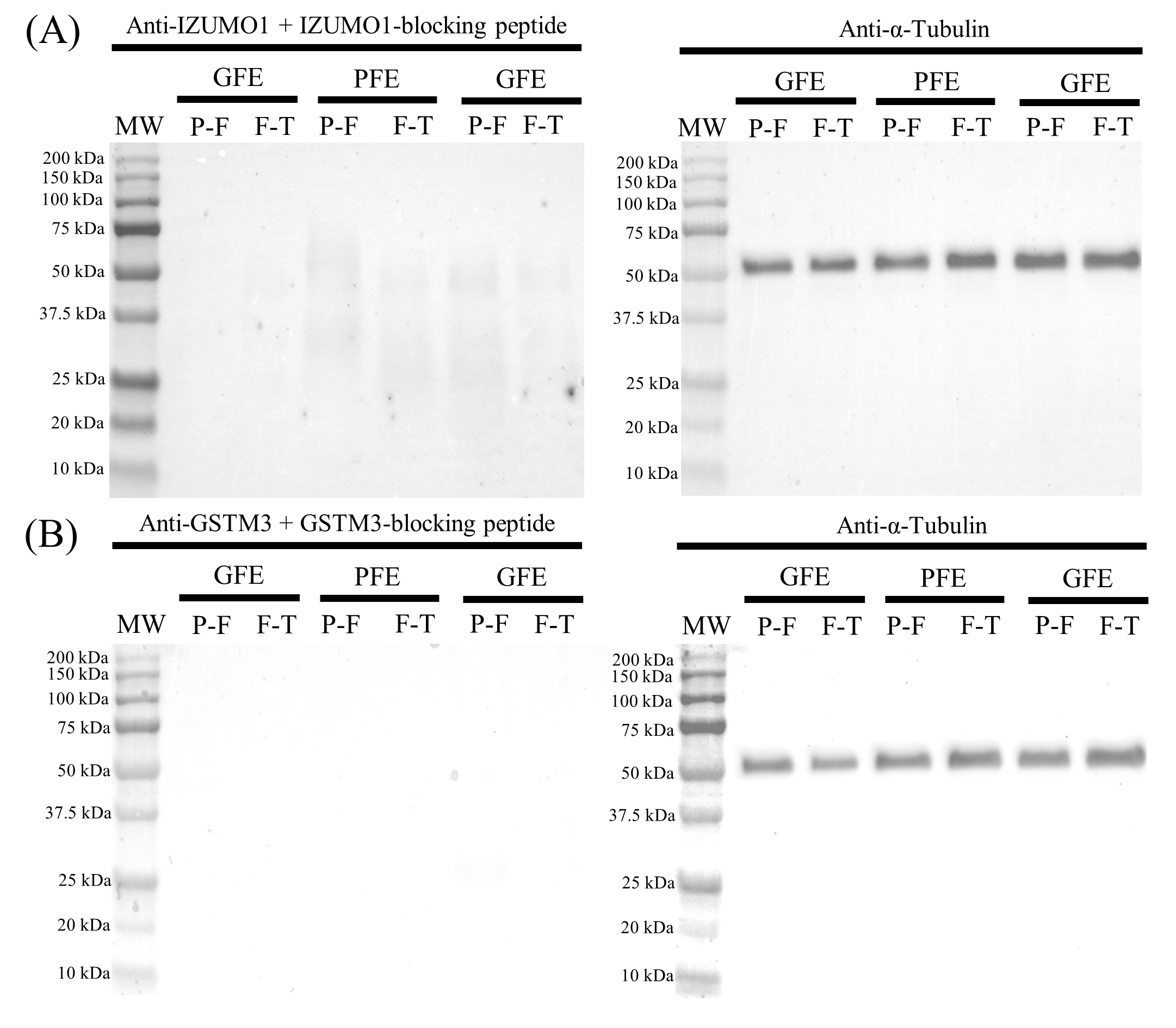

Supplement: Supplementary file 2 — Western blots resulting from incubation with the (A) IZUMO1-antibody together with the IZUMO1-blocking peptide (IZUMO1 – blocking peptide) and its loading control (α-tubulin); and (B) GSTM3-antibody with GSTM3-blocking peptide (GSTM3 – blocking peptide) and its loading control (α-tubulin). Lanes P-F: pre-frozen sperm. Lanes F-T: frozen-thawed sperm. Lanes GFE: good freezability ejaculates. Lanes PFE: poor freezability ejaculates. (TIF 1214 kb) [file 40104_2019_370_MOESM2_ESM.tif]

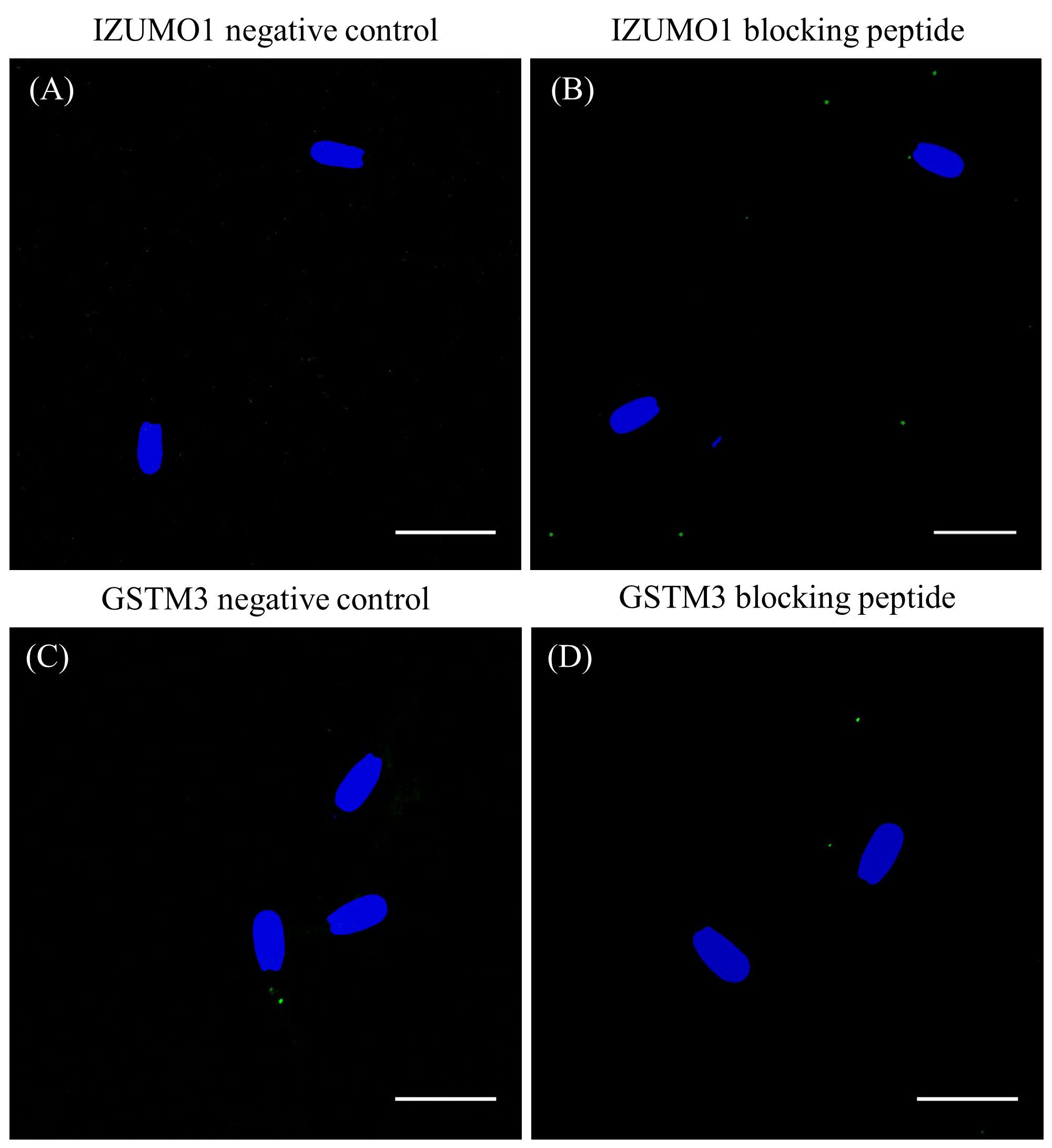

Supplement: Supplementary file 3 — Immunofluorescence of (A) IZUMO1 negative control; (B) IZUMO1-antibody incubation with the IZUMO1-blocking peptide; (C) GSTM3 negative control and (D) GSTM3-antibody incubation with the GSTM3-blocking peptide. Nucleus is shown in blue (DAPI). Scale bars: A-B: 18 μm; C-D: 14 μm. (TIF 514 kb) [file 40104_2019_370_MOESM3_ESM.tif]

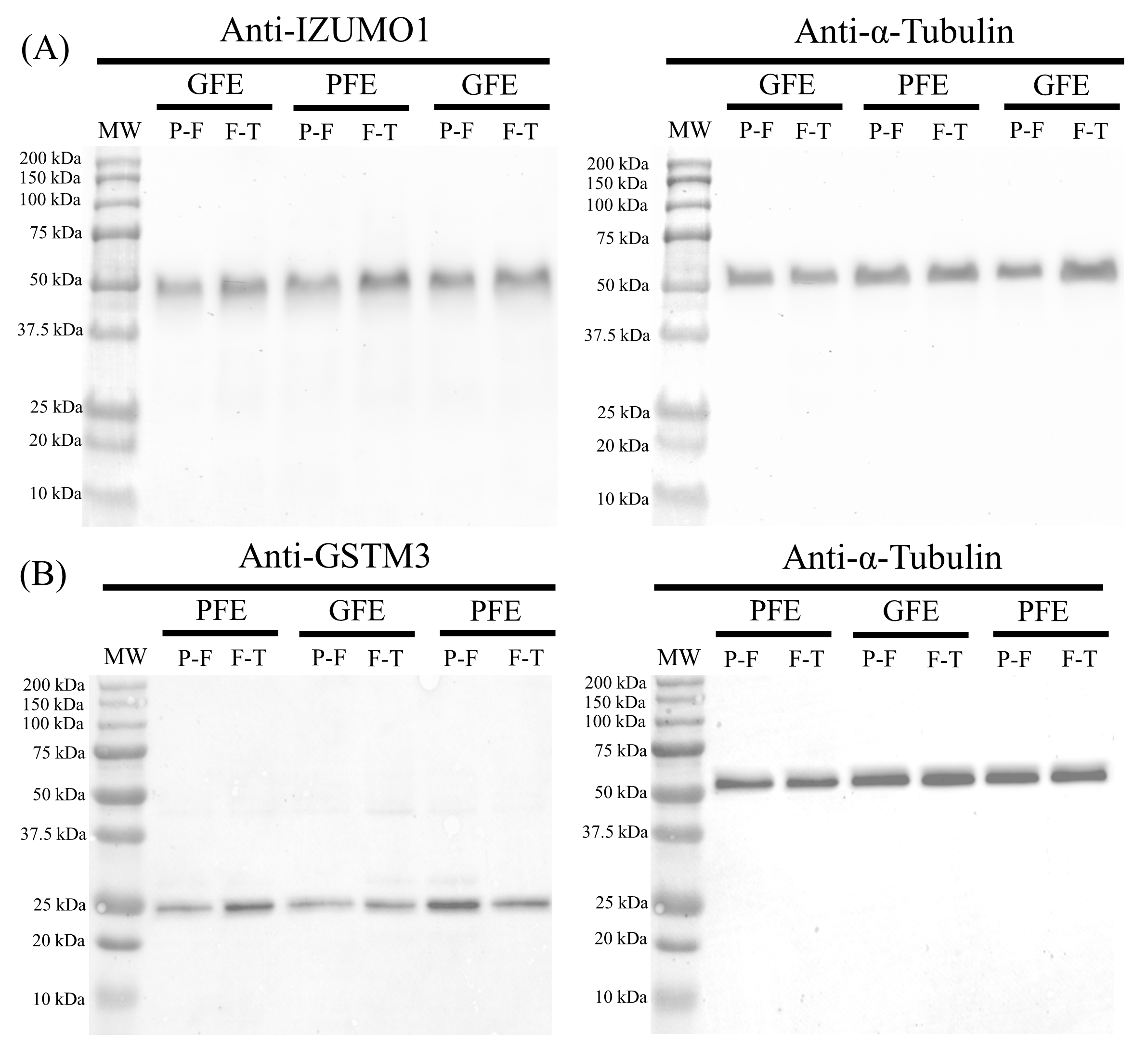

Supplement: Supplementary file 4 — Representative Western blot resulting from incubation with the (A) IZUMO1 antibody and its loading control (α-tubulin) and (B) GSTM3 antibody and its loading control (α-tubulin). Lanes P-F: pre-frozen sperm. Lanes F-T: frozen-thawed sperm. Lanes GFE: “good” freezability ejaculates. Lanes PFE: “poor” freezability ejaculates. (TIF 1479 kb) [file 40104_2019_370_MOESM4_ESM.tif]
